# Using the Framework for Reporting Adaptations and Modifications-Expanded (FRAME) to study adaptations in lung cancer screening delivery in the Veterans Health Administration: a cohort study

**DOI:** 10.1186/s43058-022-00388-x

**Published:** 2023-01-12

**Authors:** Thomas E. Strayer, Lucy B. Spalluto, Abby Burns, Christopher J. Lindsell, Claudia I. Henschke, David F. Yankelevitz, Drew Moghanaki, Robert S. Dittus, Timothy J. Vogus, Carolyn Audet, Sunil Kripalani, Christianne L. Roumie, Jennifer A. Lewis

**Affiliations:** 1 Veterans Health Administration-Tennessee Valley Healthcare System, Geriatric Research, Education and Clinical Center (GRECC), Nashville, TN USA; 2 Center for Clinical Quality and Implementation Research, Vanderbilt University Medical Center, Nashville, TN USA; 3 Department of Radiology and Radiological Sciences, Vanderbilt University Medical Center, Nashville, TN USA; 4 Vanderbilt-Ingram Cancer Center, Nashville, TN USA; 5 Veterans Health Administration-Atlanta Veterans Affairs Medical Center, Atlanta, GA USA; 6 Department of Biostatistics, Vanderbilt University School of Medicine, Nashville, TN USA; 7 Department of Radiology, Icahn School of Medicine at Mount Sinai, New York, NY USA; 8 Veterans Health Administration - Phoenix VA Health Care System, Phoenix, AZ USA; 9 Veterans Health Administration - Greater Los Angeles Veterans Affairs Medical Center, Los Angeles, CA USA; 10 Department of Radiation Oncology, University of California, Los Angeles, Los Angeles, CA USA; 11 Division of General Internal Medicine and Public Health, Vanderbilt University Medical Center, Nashville, TN USA; 12 Owen Graduate School of Management, Vanderbilt University, Nashville, TN USA; 13 Department of Health Policy, Vanderbilt University Medical Center, Nashville, TN USA; 14 Veterans Health Administration-Tennessee Valley Healthcare System, Geriatric Research, Education and Clinical Center (GRECC) and Medicine Service, Nashville, TN USA; 15 Division of Hematology/Oncology, Department of Medicine, Vanderbilt University Medical Center, 2525 West End Ave, Suite 1200, Nashville, TN 37203 USA

**Keywords:** FRAME framework, Lung cancer screening, Adaptations, Scaled implementation, Database management, COVID-19

## Abstract

**Background:**

Lung cancer screening is a complex clinical process that includes identification of eligible individuals, shared decision-making, tobacco cessation, and management of screening results. Adaptations to the delivery process for lung cancer screening in situ are understudied and underreported, with the potential loss of important considerations for improved implementation. The Framework for Reporting Adaptations and Modifications-Expanded (FRAME) allows for a systematic enumeration of adaptations to implementation of evidence-based practices. We applied FRAME to study adaptations in lung cancer screening delivery processes implemented by lung cancer screening programs in a Veterans Health Administration (VHA) Enterprise-Wide Initiative.

**Methods:**

We prospectively conducted semi-structured interviews at baseline and 1-year intervals with lung cancer screening program navigators at 10 Veterans Affairs Medical Centers (VAMCs) between 2019 and 2021. Using this data, we developed baseline (1st) process maps for each program. In subsequent years (year 1 and year 2), each program navigator reviewed the process maps. Adaptations in screening processes were identified, documented, and mapped to FRAME categories.

**Results:**

We conducted a total of 16 interviews across 10 VHA lung cancer screening programs (*n*=6 in year 1, *n*=10 in year 2) to collect adaptations. In year 1 (2020), six programs were operational and eligible. Of these, three reported adaptations to their screening process that were planned or in response to COVID-19. In year 2 (2021), all 10 programs were operational and eligible. Programs reported 14 adaptations in year 2. These adaptations were planned and unplanned and often triggered by increased workload; 57% of year 2 adaptations were related to the identification and eligibility of Veterans and 43% were related to follow-up with Veterans for screening results. Throughout the 2 years, adaptations related to data management and patient tracking occurred in 60% of programs to improve the data collection and tracking of Veterans in the screening process.

**Conclusions:**

Using FRAME, we found that adaptations occurred primarily in the areas of patient identification and communication of results due to increased workload. These findings highlight navigator time and resource considerations for sustainability and scalability of existing and future lung cancer screening programs as well as potential areas for future intervention.

**Supplementary Information:**

The online version contains supplementary material available at 10.1186/s43058-022-00388-x.

Contributions to the literature

- This study represents an example of tailoring the FRAME framework to report adaptations in lung cancer screening delivery across 10 medical centers participating in an Enterprise-Wide Initiative in the Veterans Health Administration.
- Lung cancer screening programs most frequently adapted their processes of identifying eligible individuals for screening and communicating results to patients due to the increased workload of navigators.
- We report adaptations in lung cancer screening software used to manage and track patients in the Veterans Health Administration.

## Background

Over the past two decades, implementation science has facilitated the translation of evidence-based practices (EBPs) into clinical practice with goals of fidelity, scalability, and effective dissemination [1]. Over 150 implementation frameworks exist to guide the uptake and delivery of EBPs into clinical practice. Once an evidence-based practice (EBP) is implemented, adaptations or changes in the delivery process or within the EBP itself occur that may have an impact on intervention (effect) outcomes [2, 3]. For example, a study by Fernandez et al. implemented a community-based cervical cancer screening program to low-income Hispanic women in Texas [4]. The program used small media materials combined with lay health workers and navigation to encourage and connect women with resources for Papanicolaou screening and human papillomavirus virus vaccination. Due to environmental differences and needs of the local community, adaptations were made to translate the intervention from a rural to an urban setting to increase the delivery of services and reach of the program. Other studies have adapted colorectal cancer screening interventions to different populations and have seen increases in effectiveness and maintenance outcomes (screening completion, adherence) [5, 6].

The Framework for Reporting Adaptations and Modifications-Expanded (FRAME) framework was created to guide the systematic enumeration of adaptations in implementation. The intent is to ensure sufficient documentation and description of adaptations so that they are informative to those overcoming implementation barriers and developing implementation strategies. FRAME highlights (1) *when* an adaptation occurs, (2) whether the adaptations were *planned*, (3) *who* decided to make the adaptations, (4) the *adaptation*, and (5) the *phase of intervention delivery* in which the adaptation occurred. By reporting at this detailed level, future adopters are able to evaluate the flexibility of an EBP or the delivery of the EBP and to evaluate adaptations that have been made when planning implementation or delivering the EBP in their organization [7].

Adaptations are often overlooked yet should be considered the foundation for translation of EBPs across health systems [7, 8]. The importance of adaptations can be further considered for two reasons: (1) they highlight the difficulties of maintaining fidelity within real-world clinical settings and (2) adaptations can help to achieve scalability and sustainability of EBPs across organizations [9–11]. It is unknown which steps in the lung cancer screening delivery process are most frequently adapted, how adaptations are made, and reasons for adaptations. This information could aide current and future lung cancer screening programs to plan or adapt delivery processes to meet local needs or overcome challenges. Therefore, the aim of this study was to use the FRAME framework to systematically identify and evaluate adaptations in lung cancer screening delivery within 10 Veterans Health Administration (VHA) lung cancer screening programs [7].

## Methods

### Study design

**Fig. 1:**
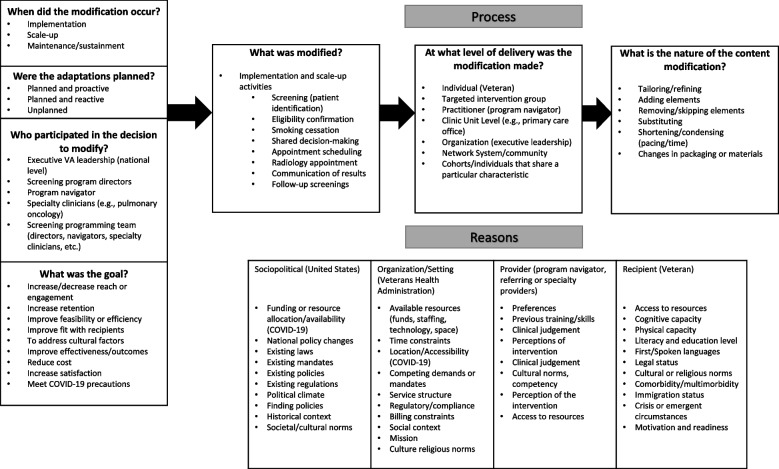
Modified FRAME to evaluate adaptations in lung cancer screening delivery FRAME stands for Framework for Reporting Adaptations and Modifications-Expanded [7]

We conducted a prospective cohort among lung cancer screening programs to collect annual adaptations (primary outcome) in the delivery of lung cancer screening using semi-structured interviews based on FRAME (Fig. 1). Time was the exposure variable with interviews conducted at baseline in 2019 or 2020 and every 12 months thereafter for two years in 2020 and 2021. Following each interview, a process map was updated to reflect the program’s reported adaptation(s).

### Setting

We included programs that were established by the VHA Enterprise-Wide Initiative (EWI), Veterans Affairs Partnership to increase Access to Lung Screening (VA-PALS), and received funding for program navigators by the Veterans Affairs (VA) Office of Rural Health. Veterans Affairs medical centers (VAMCs) that were actively enrolling patients in other lung cancer trials or had expressed interest in participating in the future were invited to join VA-PALS. Seven sites were identified through this method. An additional three sites were identified via word of mouth through lung cancer screening champions. The VA-PALS sites included Atlanta, Chicago-Hines, Cleveland, Denver, Indianapolis, Milwaukee, Nashville, Philadelphia, Phoenix, and St. Louis VAMCs [12]. The VAMCs were similar in patient volume, patient case types, number/type of clinical services, presence/size of residency programs, and research performed, and represent facilities with the high levels of clinical volumes and infrastructure relative to other facilities (VHA complexity-level classification: level 1A or 1B facilities) [13–15]. Each site hired its program navigator and initiated lung cancer screening at different times between 2017 and 2020.

### Evidence-based practice: lung cancer screening

Multiple studies including two large, randomized clinical trials have found that annual lung cancer screening with low-dose computed tomography (LDCT) reduced lung cancer-specific mortality in a high-risk population (those who are older and have extensive smoking histories) [16–19]. In 2013, the US Preventive Services Task Force (USPSTF) issued a grade B recommendation for lung cancer screening in individuals who currently or formerly smoked cigarettes (quit within the last 15 years) with a 30 pack-year history of cigarette smoking and between the ages of 55 and 80. A grade B recommendation means that clinicians should offer the service and it is covered by private insurance as a preventive health service under the Affordable Care Act [20, 21]. USPSTF expanded its eligibility criteria in 2021 to a younger population (starting at age 50) with less smoking history (20 pack-years). Individuals should also be asymptomatic and able to undergo curative treatment (surgery or radiation) [20, 22]. Since 2015, lung cancer screening has been covered by the Centers for Medicare and Medicaid Services in a similar population and this coverage decision was recently updated in 2022 [23, 24].

### Intervention: lung cancer screening programs

**Fig. 2:**
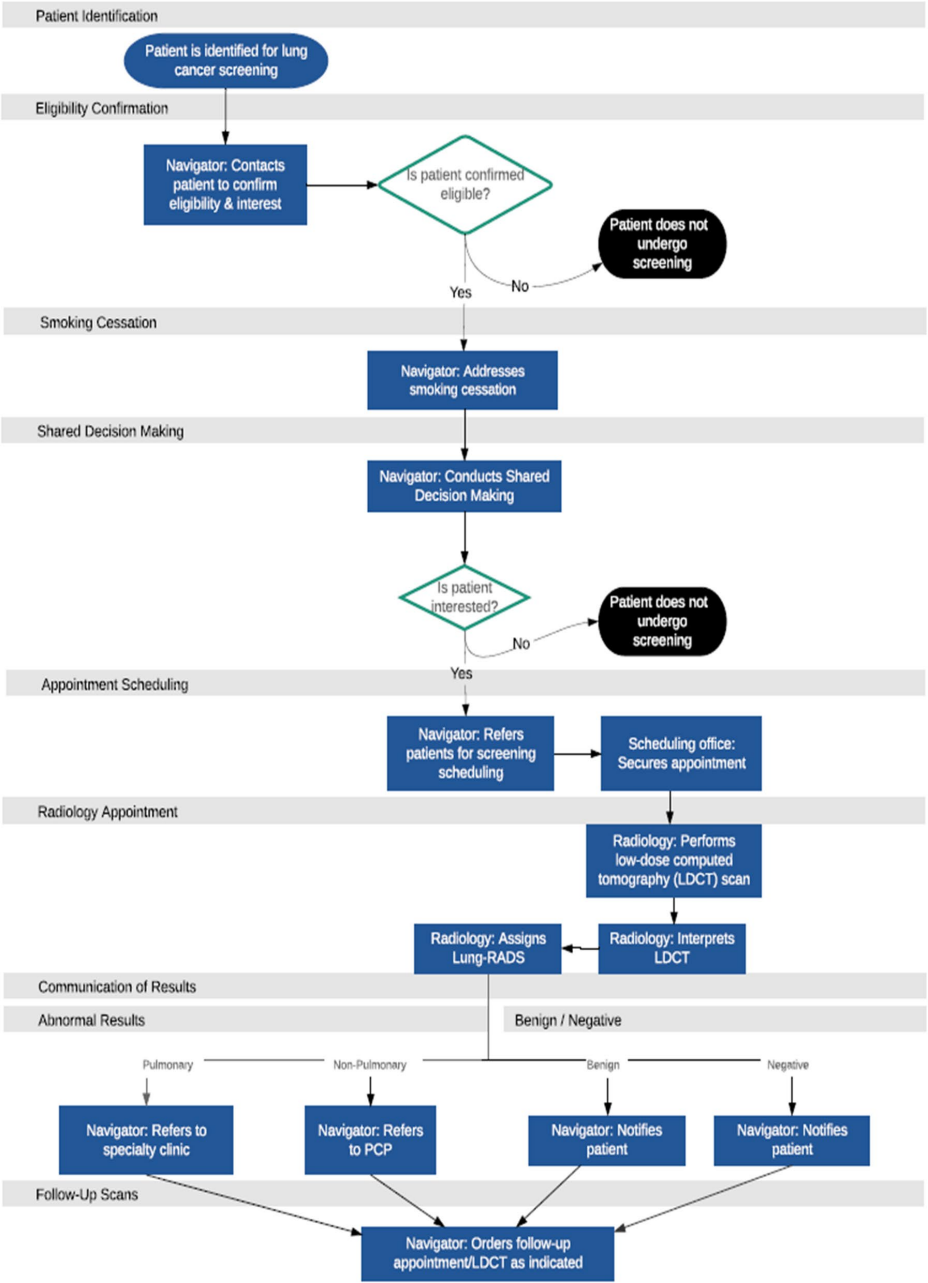
Generic lung cancer screening delivery processes at VA-PALS sites Veterans Health Administration follows U.S. Preventive Services Task Force Lung Cancer Screening eligibility criteria. VA-PALS stands for VA Partnership to increase Access to Lung Screening and implemented lung cancer screening programs at 10 VA medical centers from 2017-2021. Lung-RADS stands for Lung CT Screening Reporting & Data System and is a standardized reporting system for lung cancer screening results and management recommendations

Centralized lung cancer screening programs have been shown to increase the uptake of high-quality lung cancer screening and improve clinical outcomes [25, 26]. Starting in 2017, VA-PALS implemented lung cancer screening programs for high-risk Veterans at 10 VAMCs. Each program provides lung cancer screening with LDCT [12, 27, 28]. Programmatic resources included a program navigator, CT quality control calibration tools, navigator training, a support network, and program management software. Program navigators were responsible for conducting and coordinating care throughout the entirety or a portion of the lung cancer screening process; this is similar in concept to “delivery personnel” from the Consolidated Framework for Implementation Research. Each program had at least one program navigator, though some hired or re-hired more than one. Each program was led by a site director and was allowed to design their own screening processes based on local resources, environmental contexts, and stakeholder engagement [12]. Generalized processes for a VA-PALS lung cancer screening program are outlined in Fig. 2 and can be categorized into the following: (1) Identification and eligibility, (2) Smoking cessation, (3) Shared decision-making, (4) LDCT with interpretation, (5) Result communication, and (6) Data management/Veteran tracking.

The process begins with the identification of eligible Veterans who, if agreeable, are referred to the lung cancer screening program. Once referred to the program, a shared decision-making encounter occurs between the program navigator and the Veteran. If the Veteran agrees to screening, an LDCT exam is ordered. Smoking cessation services (education, counseling, and/or medications) are incorporated into the screening process during the shared decision-making encounter for individuals who currently smoke cigarettes. The Veteran undergoes the LDCT screening, and the imaging is reviewed and interpreted by a radiologist. Screening results are sent to the navigator who communicates them to the Veteran. The navigator takes appropriate next steps based on screening results, which can include annual repeat screening if the screening is negative, a short-term interval follow-up exam for indeterminant screenings, or a referral for further evaluation of findings concerning for lung cancer.

For program data management, each program initially used a Microsoft Excel spreadsheet for the management and tracking of Veterans throughout the screening process. The VA-PALS software development team deployed the Early Lung Cancer Action Program (ELCAP) software system for use within VHA as an open-source tool called VAPALS-ELCAP Management System [27, 28]. VAPALS-ELCAP facilitates patient tracking, data aggregation, and prospective reporting of the entire lung cancer screening process, from patient identification, shared decision-making, screening results, patient adherence, procedures performed, and cancers diagnosed.

### Study population

We conducted interviews with all of the program navigators at VA-PALS VAMCs at the start of program operations and every 12 months thereafter from 2019 to 2021.

### Data collection

Interviews to collect adaptations were conducted with Microsoft Teams and were 60 min in length. At the initial interview to assess adaptations, a team member (AB) inquired about navigator professional training, start date of screening program activities, number and history of navigators at the program, and program members. At each interview to assess adaptations, the interviewers (AB/TS) presented the program’s prior process map to the navigator for their review and the navigator walked through their processes from the beginning to the end of the current screening workflow of their program. Navigators discussed resources available that impacted workflow. Each navigator was specifically probed about process adaptations using the FRAME framework (Supplemental Table 1). Electronic notes were recorded for all interviews by two study team members [AB/TS]. A study team member (AB/TS) incorporated notes and used Lucidchart© to create process maps that outline clinical workflow, the setting, target population, and screening program team members (e.g., program navigators, clinical providers, radiologists.). We sent each program’s process map to the navigator after each interview cycle for their review, editing, and approval. After approved by each navigator, the process map was considered final for that interview cycle.

### Analysis: adaptation categorization

We defined adaptations as interval workflow process changes that impacted lung cancer screening delivery at any point along the delivery continuum depicted in Fig. 2 [7, 29]. Two team members [TS, AB] performed a content analysis of final process maps using the FRAME framework. A third investigator [JL] independently repeated this categorization. After each coding, the investigators checked for inconsistencies and any disagreement in categorization of each adaptation (< 10% of responses). All disagreement was resolved by discussion until a consensus was reached for each adaptation. The adaptations reported are from year 1 (2020) and year 2 (2021).

This program evaluation is approved by VA Central Institutional Review Board (C-IRB E19-05) and the VA Tennessee Valley Healthcare System Research & Development Committee. Committee approval was granted on November 21, 2019. The VA Organizational Assessment Subcommittee approved the study on October 1, 2019, and the VA Office of Labor and Management Relations (national union approval) approved the study on January 27, 2020. The decision to publish was made by the study team.

## Results

We conducted 16 interviews to assess adaptations, one each year (2020 and 2021), among 10 VHA lung cancer screening programs; none of the navigators refused an interview. Due to later start dates at sites B, E, and F and temporary closing of the program at site H, four programs had only one follow-up interview after baseline during the study time period (Table 1 and Supplemental Table 2). Team members that comprised each program and interview participation are provided in Supplemental Table 2. Most navigators were female (83%) and Advanced Practice Providers (75% nurse practitioners, 25% registered nurses) (Supplemental Table 3). There were three adaptations reported in 2020 and 14 adaptations reported in 2021, for a total of 17 adaptations across the 2 years (Table 1).

**Table 1:** Adaptations in Lung Cancer Screening Delivery by Program Site and Year Utilizing FRAME

Location	Year	Modification planned?	Who made the decision to modify?	Modification goal	What process was modified?	At what level of delivery?	Nature of modification	Reasons for adaptations	Baseline	Modification description
Site A	2020	Planned and reactive	Executive leadership	Meet COVID-19 precautions	Shared decision-making and smoking cessation	Patient and practitioner (program navigator)	Substituting	Organization/setting competing demands and mandates	Navigator meets with Veteran in-person for smoking cessation/shared decision-making	Navigator uses virtual/telehealth for shared decision-making/smoking cessation
	2021	Unplanned	Program site director and specialty clinician (pulmonary chief)	Improve feasibility or efficiency	Patient identification and eligibility confirmation	Individual practitioner (program navigator)	Tailoring/refining	Provider resources (time)	Navigator receives screening consult, verifies Veteran’s eligibility from chart, and checks for duplicate consult. Navigator meets with Veteran to initiate screening process	1. Medical support assistant receives consults and checks age and for duplicate consults 2. Medical support assistant schedules Veteran to meet with navigator for eligibility determination
Site B	2020	Not eligible^a^								
	2021	Unplanned	Program navigator and program site directors	Increase, decrease Reach or engagement	Patient identification and eligibility confirmation	Patient and practitioner (program navigator)	Tailoring/refining	Resources (technology)	The navigator uses the VA’s Corporate Data Warehouse^b^ to identify Veterans for screening	No longer identifying eligible Veterans through the VA’s Corporate Data Warehouse Site is now using VA’s National Lung Cancer Screening Care Platform software to conduct screening and data tracking
	2021	Unplanned	Program navigator and program site directors	Feasibility	Communication of results	Patient and practitioner (program navigator)	Removing elements	Provider resources (time)	Navigator calls Veterans when screening results are negative/benign	Communication of results via mailed letter to Veterans for negative/benign screening results
Site C	2020	No reported changes								
	2021	Unplanned	Screening program team	Decrease reach or engagement	Patient identification and eligibility confirmation	Patient and practitioner (program navigator)	Removing elements	Sociopolitical (funding or resource allocation/availability) Provider resources (time)	Navigators send letters to all eligible Veterans for recruitment	Program no longer sends letters to Veterans for recruitment
Site D	2020	Planned and reactive	Executive leadership	Meet COVID-19 precautions	Shared decision-making and smoking cessation	Patient and practitioner (program navigator)	Substituting	Organization/setting competing demands and mandates	Navigator meets with Veteran in-person for group shared decision-making/smoking cessation	1. Navigators use virtual/telehealth for shared decision-making/smoking cessation 2. Temporarily stopped referral process 3. Changes made to scheduling in-person visits for COVID-19 precautions
	2021	Planned and reactive	Screening program team	Improve feasibility or efficiency	Patient identification and eligibility confirmation	Patient and practitioner (program navigator) and clinic unit level	Shortening/condensing	Provider resources (time)	Navigator responsible for eligibility verification (pack year calculation) of Veterans in the screening program	Lung cancer screening (LCS) consult modified to include tobacco pack year calculator and require referring provider to enter pack years and confirm eligibility
Site E	2020	Not eligible^a^								
	2021	Planned and reactive	Program navigator and program site directors	Increase engagement	Screening and eligibility confirmation	Patient and practitioner (program navigator) and clinic unit level	Adding elements	Organization/setting (location/accessibility)	Veterans only identified and enrolled if the navigator recruits from primary care clinics	Tobacco cessation program and pulmonary department trained and refer to lung cancer screening program
	2021	Planned and reactive	Program navigator and program site directors	Increase engagement (primary care)	Communication of results	Patient and practitioner (program navigator) and clinic unit level	Removing elements	Provider preferences	Primary care alerted to all screening results	Primary care no longer alerted with negative/benign screening results
Site F	2020	Not eligible^a^								
	2021	Planned and reactive	Screening program team	Increase engagement	Patient identification	Patient and practitioner (program navigator) and clinic unit level	Adding elements	Organization/setting (location/accessibility)	Primary care is the main clinic to refer Veterans to lung cancer screening	Partnered with infectious disease to increase referrals of all eligible Veterans
	2021	Planned and reactive	Screening program team	Improve feasibility or efficiency	Communication of results	Patient and practitioner (program navigator)	Substituting	Provider resources (time)	Navigator sends letters to all Veteran with screening results.	1. Changed results communication process to eliminate mailing 2. Add phone call to Veteran for all screening results
Site G	2020	Planned and reactive	Executive leadership	COVID-19 precautions	Shared decision-making and smoking cessation	Patient and practitioner (program navigator)	Substituting	Organization/setting competing demands and mandates	Navigator meets with Veteran in-person for shared decision-making and smoking cessation	Stopped in-person shared decision-making and smoking cessation visits
	2021	Unplanned	Navigator	Improve feasibility or efficiency	Communication of results	Patient and practitioner (program navigator)	Substituting	Provider resources (time)	Navigator calls Veterans when screening results are negative/benign	Communication of results via mailed letter to Veterans with negative/benign screening results
Site H	2020	No navigator^a^								
	2021	Planned and reactive	Screening program team	Improve feasibility or efficiency	Follow-up of screening results	Patient and practitioner (program navigator)	Tailoring/refining	Provider resources (time)	Navigator uses the date the screening is scheduled and checks electronic health record to see if completed	Navigator receives an alert within electronic health record when screening is completed. Avoids delays in communication of results
Site I	2020	No reported changes								
	2021	No reported changes								
Site J	2020	No reported changes								
	2021	Unplanned	Navigator	Improve feasibility or efficiency	Patient identification, eligibility confirmation	Patient	Substituting	Provider resources (time)	Navigator calls Veteran eligible for screening	Navigator sends letters to Veterans eligible for screening
	2021	Planned and proactive	Research stakeholder and collaboration group	Improve feasibility or efficiency	Communication of results	Patient and practitioner (program navigator)	Tailoring/refining	Organization/setting competing demands and mandates	Navigator waits for recurring meetings to discuss non-definitive screening results with providers.	Unclear screening results are discussed in a new lung nodule conference that occurs weekly.
	2021	Planned	Navigator	Improve feasibility or efficiency	Follow-up	Patient	Tailoring/refining	Provider resources (time)	Navigator schedules 6-month and 12-month follow-up and 3 months post-initial screening	Navigator schedules 3 months and 6 months at initial screening and 12-month and 3-month follow-up

*COVID-19* coronavirus disease of 2019, *EMR* electronic medical record

^a^Programs at sites B, E, and F were not eligible for adaptations as their program became operational in 2020; baseline process maps were developed this year. The program at site H was without a navigator at the time of interviews. However, the screening program was on-going

^b^The Veteran Affairs Corporate Data Warehouse is the national database comprised of data obtained from the electronic health record system.

### Year 1 adaptations (2020)

Six programs were operational and eligible for adaptations. Four sites reported no adaptations. Three sites reported “top down” adaptations by organizational leadership that were planned, reactive, and due to the coronavirus disease of 2019 (COVID-19) pandemic. These included social distancing and reduction of in-person activities for shared decision-making and smoking cessation services. Adaptations included telehealth or virtual encounters for eligibility and shared decision-making, but only three programs needed to adapt in this manner; many were already utilizing means of communication and scheduling that adhered to social distancing guidelines during the COVID-19 pandemic.

### Year 2 adaptations (2021)

All 10 programs were operational, and nine of the 10 programs reported at least one adaptation. There were a total of 14 adaptations reported. Eight adaptations (57%) occurred in the patient identification and eligibility confirmation. Six adaptations (43%) were related to result communication and follow-up with Veterans. All adaptations were decided upon by a combination of the screening team including program leadership and/or navigators. Most adaptations (64%) were due to constrained resources (navigator time). Activities such as reaching Veterans via mail or on the phone at a time convenient for the Veteran, verifying eligibility, and letting Veterans know the next steps in the screening process can take considerable time. To alleviate these resource constraints, navigators reported that programs added personnel, contacting Veterans via different mechanisms (phone to mail or vice versa), or even reducing recruitment. These adaptations were unplanned and “internal decisions” made between the site director(s) and the navigator in 2021.

### Data management and Veteran tracking adaptations

All programs began with an Excel spreadsheet, and six of the ten (60%) noted adaptations in their data management system (Table 2). One program began using the VAPALS-ELCAP Management System (10%), three (30%) programs began using the VA National Lung Cancer Screening Platform, one (10%) program used a REDCAP variation, and one (10%) program used LungView©. Reported reasons for adaptation included navigator time constraints to enter data and improved tracking of Veterans.

**Table 2:** Data management adaptations by site

Location	Baseline data tool	Year change occurred	New data tool	Who made the decision to use new tool?	Goal of using new data tool	Modification description
Site A	Microsoft Excel	2021	LungView©	Program directors and navigators	Improve tracking of Veterans longitudinally	Instead of Microsoft Excel, LungView© was used for its ability to communicate with Veteran electronic medical record and improved ease in tracking
Site B	Microsoft Excel	2021	VISN 23 System (National Lung cancer Screening Care Platform Tool 1.0)	Program directors and navigators	To decrease navigator time on data entry	Instead of Microsoft Excel, the National Lung Cancer Screening Care Platform was adopted as it has the capabilities to reduce the time of navigator data entry
Site C	Microsoft Excel	No reported changes				
Site D	Microsoft Excel	No reported changes				
Site E	Microsoft Excel	No reported changes				
Site F	Microsoft Excel	2020	VISN 23 System (National Lung cancer Screening Care Platform Tool 1.0)	Program directors and navigators	Improve tracking of Veterans and screenings	Instead of Microsoft Excel, the National Lung Cancer Screening Care Platform was adopted as it has the capabilities to integrate with the electronic health record and improve collaborations with other care team members
Site G	Microsoft Excel	2020	REDCap System	Program directors and navigators	Improve tracking of Veterans longitudinally	This modification took approximately 3 months in 2020 to implement from Microsoft Excel to the VA REDCap system. The overall purpose of this change was to improve the longitudinal tracking of Veteran data
Site H	Microsoft Excel	No reported changes				
Site I	Microsoft Excel	2021	VAPALS-ELCAP Management System	Program directors and navigators	Improve tracking of Veterans and screenings	Modification was introduced during 2021. VAPALS-ELCAP is a software system designed for use in the VA for lung cancer screening. Programming is noted to be adaptable to current needs of navigators
Site J	Microsoft Excel	2021	VISN 23 System and Excel spreadsheet (National Lung cancer Screening Care Platform Tool 1.0)	Program directors and navigators	Improve tracking of Veterans longitudinally	In combination with Microsoft Excel, the National Lung Cancer Screening Care Platform was adopted as it has the capabilities to reduce time of the navigator and allow for better tracking of Veterans and reduce loss of follow-up

## Discussion

This is the first study that the authors are aware of to report adaptations in the delivery of lung cancer screening. We found that identification and determination of individuals’ eligibility for screening was the most frequently adapted step in the delivery of lung cancer screening. There were also adaptations in shared decision-making, tobacco cessation, follow-up of screening results, and communication of results to patients.

Following the publication of the NLST in 2011, lung cancer screening programs were established across the US with the goal to provide safe and effective screening to individuals at high-risk for lung cancer [30]. We have learned much over the last decade about the implementation of lung cancer screening programs. Thought leaders in the field recommend creating organizational change, incorporating “essential elements,” and establishing workflow logistics to start lung cancer screening programs [30–32]. Essential elements of screening programs include using a structured reporting system to classify screening results (i.e., IELCAP, Lung-RADS), providing patient and clinician education, planning for and hiring personnel needed to track and manage screenings, establishment of referral networks and investing in services needed to evaluate and treat early-stage lung cancer (i.e., interventional radiology, pulmonology, thoracic surgery) [30]. Program logistics are recommended to include eligibility assessment, image acquisition, image review, communication of screening results, and referring patients for evaluation and management of abnormal findings concerning for lung cancer [32]. A single-site, retrospective cohort found a centralized approach in the development of screening programs was associated with superior outcomes compared to a decentralized approach; a centralized screening program is one in which the lung cancer screening programs manages all of the delivery processes and a decentralized program manages a portion of the delivery processes [25]. Others have described how programs were established in a national healthcare system [33]. However, this is the first study to longitudinally describe and evaluate how lung cancer screening programs adapted their delivery processes over several years using process mapping.

It is not surprising that identification and assessment of eligibility was the most frequently adapted step in the delivery of lung cancer screening. It can be cumbersome because it requires obtaining and parsing complex information on smoking history such as start and stop dates and number of packs smoked per day and assessing this annually to ensure individuals remain appropriate for screening; an individual can become ineligible if their age becomes greater than 80 or the quit year of those who formerly smoked extends beyond 15 years. Furthermore, smoking histories are often not captured accurately in electronic health records, which makes this step critical in the screening process [33–35]. Lastly, eligibility assessment is a known challenge of lung cancer screening implementation [33, 36–39]. This study further highlights the challenge of identifying eligible patients through longitudinal evaluations of real-world lung cancer screening programs.

This is the first study to the authors’ knowledge that uses the FRAME framework to collect data on delivery processes of an EBP. Using the FRAME framework in this manner was feasible and helpful to systematically collect adaptations across 10 lung cancer screening programs over 2 years. This framework could be used in a similar manner to evaluate the delivery of other EBPs. Using the FRAME framework allowed detailed information that may be useful to existing programs considering making adaptations to their delivery processes. It may also be helpful for organizations planning to implement lung cancer screening programs as it highlights areas where programs could consider additional resources. This may include a medical support assistant to help with part of the eligibility assessment as done by site A or task-shifting to having primary care providers perform the eligibility assessment instead of the program navigator as in site D. Knowing and planning for these particular steps in the delivery process can help shape current and future programs. In fact, our team was frequently contacted by VA-PALS leadership to share the process maps with programs within and outside VA-PALS. Thus, the process maps became an implementation tool in and of themselves.

There have been several studies that assess adaptations of interventions to implement cancer screenings. The BeneFIT study reported adaptations in health-plan-initiated fecal immunochemical test (FIT) programs for colorectal cancer screening in Oregon and Washington State from year 1 to year 2. The team used frameworks by Stirman et al. as well as FRAME to capture their adaptations and found substantial variations in the adaptations that were made: some adaptations targeted health centers while others targeted member populations; some adapted their program outreach to a different target population; some adaptations tailored existing program components while others added components; program leadership was adapted from a local to a national scale; and personnel who administered the program also were adapted [40]. Similar to our results, these adaptations reflect adjustment programs made in response to resources and local contextual factors in order to overcome challenges that they encountered. Other cancer screening studies have made adaptations to interventions to improve program reach, effectiveness, and maintenance [4–6]. However, our study is unique in that we used the FRAME framework to evaluate adaptations in a delivery process as opposed to adaptations in an intervention. We also used process mapping as a tool to document and communicate adaptations.

Multiple factors influenced the adaptations we observed among VA-PALS lung cancer screening programs. Adaptations in the first year were mostly in response to COVID-19 to limit in-person screening-related activities. This is similar to what occurred in other cancer screening programs during the COVID-19 pandemic; adaptations were made to prioritize patients at highest risk, transition appointments from in-person to virtual, among other safety precautions [41–43]. Only a few VA-PALS lung cancer screening programs needed to adapt from in-person to virtual appointments to conduct shared decision-making as many were already reaching Veterans via virtual appointments. The adaptations related to COVID-19 were still in place in 2021 and we expect these will likely persist to some degree to reach as many Veterans as possible. Adaptations in year 2 were largely focused on time and resource constraints of the navigators. Navigators’ adaptations focused much of their time on recruitment and verification of screening eligibility, as well as communication of results back to Veterans. Adaptations in these activities focused on creating more efficient processes to reach Veterans. Interestingly, there was site-level variability in how programs approached constraints on navigator time. Some programs reduced the number of Veterans that were recruited for screening while other programs reduced points of contact for communication of results (using phone calls versus mailed letters, etc.). Only one site adapted to the increasing volume by adding sufficient navigator resources to meet demand (e.g., additional support staff).

Almost all steps in the delivery process of lung cancer screening were adaptable across the 10 programs. There was variability in available resources and in programs’ approaches to overcome barriers. At some sites, multiple navigators were able to work as a team to coordinate screening activities, while others needed additional support personnel to meet screening demand. One program noted a navigator was on-leave and an outside staff member covered screening program activities. To make communication of results to Veterans more efficient, two programs replaced telephone calls with mailed letters, while another program did the opposite. These adaptations highlight how agile programs were at adjusting workflows to fulfill their commitment and responsibilities.

Part of the VA-PALS program was the development and installation of the VAPALS-ELCAP Management System as a tool for all sites; Microsoft Excel was meant to be only a temporary tool. Barriers to software installation led to a delayed timeline, and only one program was able to use the software system during the study time period. VAPALS-ELCAP has been pilot tested and is currently managing over 1,500 Veterans in a centralized screening program. Since the time of data collection for this study, the tool has been installed at a second program. Several programs adapted to a data management tool used by other VAMCs (National Lung Cancer Screening Care Platform Tool 1.0). All adapting programs cited efficiency as a reason for adaptation. A target of future work may aim to address the step-by-step decision-making pathway for data management tools for future implementation.

### Implications

Delivery processes of EBPs are often adapted, but how and why remains unexplored, especially in nationally scaled programs [8]. The results of this work highlight variations in lung cancer screening delivery and adaptations to overcome process barriers from a national perspective, often highlighting resource constraints as a reason for adaptations, particularly program navigator time. The results also demonstrate that local process variation and adaptations persist even in highly integrated systems. Understanding the complexity of widely scaled pragmatic interventions should support the need for developers to consider and promote adaptations in pragmatic trials. In addition, these data show the complexity of capturing adaptations in EBP delivery processes and the utility of an established framework such as FRAME.

Understanding adaptations to the delivery of EBPs allows for more successful implementation through a better understanding of sites’ unique contextual factors. Much of the existing literature has focused on adaptations of the EBP to the local context [44–47]. For example, many studies discuss adaptations of interventions to fit their unique healthcare systems, clinical practice, and patient population [44–47]. Our work is unique in that the focus is on adaptations in delivery processes using FRAME. Accordingly, we highlight the unique constraints and needs of various sites when implementing the same EBP across 10 VAMCs. Each site was given the opportunity to learn from practice evolution, which is a core component of identifying delivery processes for broader adoption. These findings highlight that implementation of EBPs is not static and processes where adaptations were common (identifying eligible patients and communication of screening results to patients) may be key to scalability and sustainability over time.

### Limitations

This study has several limitations. We are unable to assess adaptations completed prior to 2020 or for programs that were not fully operational or had just become operational in year one. Additionally, adaptation reporting relied on program navigators. Reporting bias may exist as programs may both under-report adaptations and over-report adaptations. For each assessed adaptation, we are unable to assess fidelity to the adaptation or the clinical impact of these adaptations on screening outcomes. Furthermore, we evaluated adaptations from process maps reviewed by the navigator at each program; other perspectives were not obtained. There may have been adaptations to the screening processes of which navigators were unaware. Our process maps were shared via paper copies with all VA-PALS lung cancer screening programs at a VA-PALS in-person conference in 2020 as a resource, and this may have influenced adaptations we observed in 2021. Finally, programs within and outside VHA develop delivery processes (identify eligible screening candidates, perform shared decision-making, tobacco treatment, follow up and communicate screening results, etc.) for lung cancer screening. However, this study focused on the VHA healthcare system and included facilities with high clinical volumes and existing infrastructure. Therefore, our findings may or may not be generalizable to other healthcare systems or facility types.

## Conclusion

Using FRAME, we found that most programs made adaptations to components of the lung cancer screening delivery process, most frequently in the area of patient identification and determination of eligibility. Programs also made several adaptations in their lung cancer screening management system to improve the efficiency of patient tracking. These adaptations highlight challenges encountered and may inform current, and future, lung cancer screening program implementation, especially scalability and sustainability. Future directions include the determination of the impact adaptations have on the effectiveness of lung cancer screening programs, namely core functions such as recruitment, shared decision-making, and follow-up.

## Supplementary Information

**Additional file 1: Supplemental Table 1.** Interview Guide. **Supplemental Table 2.** Lung Cancer Screening Program Team Members and Process Map Interview Participation. **Supplemental Table 3.** Navigator Characteristics.

## Data Availability

All relevant data are within the manuscript and its supporting information files. The datasets used and/or analyzed during the current study are available from the corresponding author on reasonable request. This includes initial and updated process maps for each site location.

## Abbreviations

COVID-19: Coronavirus disease of 2019
FRAME: Framework for Reporting Adaptations and Modifications-Expanded
VA: Veteran Affairs
VA-PALS: VA-Partnership to increase Access to Lung Screening
VHA: Veterans Health Administration
VAMC: Veterans Affairs Medical Centers
EBP: Evidence-based practice
USPSTF: US Preventive Services Task Force
EWI: Enterprise-Wide Initiative
LDCT: Low-dose computed tomography
ELCAP: Early Lung Cancer Action Program
