# Supplementary material for: Using the Framework for Reporting Adaptations and Modifications-Expanded (FRAME) to study adaptations in lung cancer screening delivery in the Veterans Health Administration: a cohort study
Source: Implement Sci Commun. 2023 Jan 12;4:5. doi: 10.1186/s43058-022-00388-x (PMC9836333; doi:10.1186/s43058-022-00388-x)
Supplement: Supplementary file 1 — Additional file 1: Supplemental Table 1. Interview Guide. Supplemental Table 2. Lung Cancer Screening Program Team Members and Process Map Interview Participation. Supplemental Table 3. Navigator Characteristics. [file 43058_2022_388_MOESM1_ESM.docx]

Additional file

Supplemental Table 1: Interview Guide

| Concept | Interview Guide Item |
| --- | --- |
| Navigator Start Date* | What was the month and year you started as a lung cancer screening program navigator? |
| Work Week | How many days per week do you work as the lung cancer screening program navigator? |
| Navigators per program | How many navigators are at your site for the lung cancer screening program? Were there any previous navigators? If so, how many and how long did they serve in this role? |
| Navigator training | Have you gone through navigator training with Mt. Sinai? |
| Initial Program processes  (baseline interview) | Tell me how Veterans are screened at your VA?   - How are Veterans identified? Who identifies them? (i.e. how Veterans enter the program) [measure: program entry] - How is eligibility confirmed? Who confirms eligibility? - Who conducts shared decision making? How is shared decision making performed (telephone/in-person)? - How is scheduling performed? Who schedules the Veteran? - How is smoking cessation addressed? Who performs this? - Who orders the screening? - Does screening take place on the same day as the shared decision making? - What system does your radiologist use to categorize screening results (Lung-RADs, I-ELCAP, etc.) - Who receives the screening results? - Who communicates results to patient? How does the patient receive screening results? Who communicates results to the patient’s primary care provider? - How are abnormal results handled? - How are annual follow up exams tracked? Who tracks Veterans needing follow up/annual screening? - How are you tracking the percentage of rural Veterans you screen? How do you determine a Veteran’s rural status? Have you encountered any difficulties or challenges in screening rural Veterans? If so, what might help this? |
| Process Adaptations  (subsequent interviews) | In the past 6 months, has your screening processes changed? If so, tell me how your VA’s screening process has changed?   - When and how were modifications made? - Were these changes planned and proactive (e.g., intentional adaptation) or unplanned and reactive (e.g., in response to other forces)? - Who decided changes were necessary? - What was modified? - At what level in the system of delivery were changes made (executive leadership, department/service line leadership, healthcare provider, or staff level)? - What were the reasons modifications were made and what contextual drivers were at play? |
| Tracking & Management Tool* | What tracking and management tool were you using when your program started (commercial system, excel spreadsheet, system created by VA, etc.)?  Have you changed to a different management tool?   - When did this change occur (month and year)? - Why did you switch to a different management tool? (Who was involved in this process?) - What is the main difference between the two management tools? - How long did it take to implement this new management tool (months)? |
| Program team | Please describe who is on your team, specifically each person’s role and specialty. |

*Navigator start date was collected once at the first interview with each navigator. Tracking & Management tool was added to the 2021 interview.

Supplemental Table 2: Lung Cancer Screening Program Team Members and Process Map Interview Participation

| Site | Team Members* | Year of Baseline Process Map | Participate in Year 1 (2020) Adaptation Interview | Participate in Year 2 (2021) Adaptation Interview |
| --- | --- | --- | --- | --- |
| A | Medical Support Assistant | 2019 | yes | yes |
|  | Navigator |  |  |  |
|  | Coordinator |  |  |  |
|  | Chief of Pulmonary |  |  |  |
|  | Pulmonologist (Program Director) |  |  |  |
|  | Thoracic Surgeon |  |  |  |
| B | Oncologist (Program Director) | 2020 | Not eligible | yes |
|  | Pulmonary Chief |  |  |  |
|  | Pulmonary NP |  |  |  |
|  | Chief of Medicine |  |  |  |
|  | Navigator |  |  |  |
|  | Acting Ambulatory Chief |  |  |  |
|  | Radiology |  |  |  |
| C | Navigator | 2019 | yes | yes |
|  | Vice Chair of Medicine (Program Director) |  |  |  |
|  | Pulmonary Chief |  |  |  |
|  | Cancer Care Coordinator |  |  |  |
|  | Radiology |  |  |  |
| D | Pulmonologist (Program Co-Director) | 2019 | yes | yes |
|  | Oncologist (Program Co-Director) |  |  |  |
|  | Radiologist |  |  |  |
|  | Radiologist |  |  |  |
|  | Primary Care Physician |  |  |  |
|  | Thoracic Surgeon |  |  |  |
|  | Smoking Cessation Counselor |  |  |  |
|  | Navigator |  |  |  |
| E | Pulmonologist (Program Director) | 2020 | Not eligible | yes |
|  | Navigator |  |  |  |
|  | Radiologist |  |  |  |
| F | Pulmonologist (Program Director) | 2020 | Not eligible | yes |
|  | Pulmonologist |  |  |  |
|  | Pulmonologist |  |  |  |
|  | Radiologist |  |  |  |
|  | Radiologist |  |  |  |
|  | Thoracic Surgeon |  |  |  |
|  | Navigator |  |  |  |
|  | Radiation Oncologist |  |  |  |
| G | Navigator | 2019 | yes | yes |
|  | Pulmonologist (Program Director) |  |  |  |
|  | Radiologist |  |  |  |
| H | Oncologist (Program Director) | 2019 | No navigator | yes |
|  | Radiologist |  |  |  |
|  | Navigator |  |  |  |
|  | Pulmonologist |  |  |  |
| I | Navigator | 2019 | yes | yes |
|  | Navigator |  |  |  |
|  | Pulmonologist (Program Director) |  |  |  |
|  | Radiologist |  |  |  |
| J | Radiologist | 2019 | yes | yes |
|  | Radiologist |  |  |  |
|  | Pulmonologist (Program Director) |  |  |  |
|  | Pulmonologist |  |  |  |
|  | Navigator |  |  |  |

*These data are reported by navigator in 2020 or 2021

Supplemental Table 3: Navigator Characteristics

| Site | Clinical  Training* | Start Dates* | Work as program* navigator | Navigator Training Date* | Navigator history at each program* |
| --- | --- | --- | --- | --- | --- |
| A | APP | July 2018 | 5 days/week | October 2018 | No previous navigator |
| B | RN | May 2020 | 5 days/week | Delayed due to COVID-19 | One was hired 6 years ago and worked for 6-7 months |
| C | APP | October 2019 | 5 days/week | October 2019 | No previous navigator |
| D | APP | February 2019 | 4 days/week | March 2019 | No previous navigator |
| E | APP | July 2020 | 5 days/week | Delayed due to COVID-19 | 2 previous navigators in past two years |
| F | APP | February 2020 | 4 days/week | Delayed due to COVID-19 | No previous navigator |
| G | APP | March 2019 | 5 days/week | November 2018 | No previous navigator |
| H | APP | January 2019 & April 2021 | 5 days/week | April 2019 | First site navigator; 1 previous navigator in 2020 that worked for 6 months |
| I | RN | December 2017 | 1 day/week | Developed VA-PALS navigator training | First site and VA-PALS navigator |
|  | RN | April 2019 | 5 days/week | April 2019 | Second navigator |
| J | APP | April 2019 | 5 days/week | November 2018 | No previous navigator |

*Self-reported by navigators at interviews in 2020 or 2021; APP: advanced practice provider; RN: registered nurse
